# A Collagen-Based Scaffold for Promoting Neural Plasticity in a Rat Model of Spinal Cord Injury

**DOI:** 10.3390/polym12102245

**Published:** 2020-09-29

**Authors:** Jue-Zong Yeh, Ding-Han Wang, Juin-Hong Cherng, Yi-Wen Wang, Gang-Yi Fan, Nien-Hsien Liou, Jiang-Chuan Liu, Chung-Hsing Chou

**Affiliations:** 1 Department of Pharmacy, Tri-Service General Hospital, National Defense Medical Center, Taipei 114, Taiwan; yehjuezong@gmail.com; 2 School of Dentistry, National Yang-Ming University, Taipei 112, Taiwan; nn2399906@gmail.com; 3 Department and Graduate Institute of Biology and Anatomy, National Defense Medical Center, Taipei 114, Taiwan; i72bbb@gmail.com (J.-H.C.); christmas1035@hotmail.com (Y.-W.W.); medregenliou@yahoo.com.tw (N.-H.L.); yjcliu@ndmctsgh.edu.tw (J.-C.L.); 4 Department of Gerontological Health Care, National Taipei University of Nursing and Health Sciences, Taipei 112, Taiwan; 5 Division of Urology, Department of Surgery, Tri-Service General Hospital, National Defense Medical Center, Taipei 114, Taiwan; u9310318@gmail.com; 6 Department of Neurology, Tri-Service General Hospital, National Defense Medical Center, Taipei 114, Taiwan; 7 Graduate Institute of Medical Sciences, National Defense Medical Center, Taipei 114, Taiwan

**Keywords:** spinal cord injury, neural plasticity, axonal regeneration, collagen scaffold

## Abstract

In spinal cord injury (SCI) therapy, glial scarring formed by activated astrocytes is a primary problem that needs to be solved to enhance axonal regeneration. In this study, we developed and used a collagen scaffold for glial scar replacement to create an appropriate environment in an SCI rat model and determined whether neural plasticity can be manipulated using this approach. We used four experimental groups, as follows: SCI-collagen scaffold, SCI control, normal spinal cord-collagen scaffold, and normal control. The collagen scaffold showed excellent in vitro and in vivo biocompatibility. Immunofluorescence staining revealed increased expression of neurofilament and fibronectin and reduced expression of glial fibrillary acidic protein and anti-chondroitin sulfate in the collagen scaffold-treated SCI rats at 1 and 4 weeks post-implantation compared with that in untreated SCI control. This indicates that the collagen scaffold implantation promoted neuronal survival and axonal growth within the injured site and prevented glial scar formation by controlling astrocyte production for their normal functioning. Our study highlights the feasibility of using the collagen scaffold in SCI repair. The collagen scaffold was found to exert beneficial effects on neuronal activity and may help in manipulating synaptic plasticity, implying its great potential for clinical application in SCI.

## 1. Introduction

Spinal cord injury (SCI) is a devastating condition encountered by neurosurgeons [1]. In SCI, nerve fibers or axons of the central nervous system (CNS) are frequently unable to regenerate, resulting in neurological disabilities. The major failures of axonal regeneration after injury are attributed to scar formation, a long-lasting inflammatory response, increasing levels of proteoglycans, and demyelination [2,3,4]. Among these factors, glial scar formation is considered a primary cause of the limited axonal regenerative capability in the mammalian CNS, as it hampers the regrowth and remodeling of axons [5,6].

In general, scar formation involves a resolution phase and a tissue function restoration phase. However, the tissue scarring mechanism in the CNS is more complex than that in many other systems and often leads to incomplete tissue repair. Under the normal condition, glia cells support signal transmission and neuronal function [7]. Following SCI, glial activation is triggered in response to the mechanical damage in the subacute phase, and this leads to newly hypertrophic and proliferating reactive astrocytes forming a glial scar that densely populates the borders of the injury site, separating damaged tissue from healthy tissue [8,9,10]. The mechanical trauma further leads to injury progression by facilitating recruitment and infiltration of non-resident cells with complex downstream signaling cascade effects on neuronal function and regeneration of myelin sheath [7,11]. The glial scar represents a physical and molecular barrier to axonal development and has become an important topic for research on tissue regeneration in chronic SCI [10,12,13].

Therefore, the most important aspect to be considered for successful axonal regeneration and synapse reformation after SCI is the control of glial scar formation. Recent studies have demonstrated neuro-regeneration-targeted approaches focusing on graft engineering and pharmacological and gene therapy [14]. Biomaterial-based graft development for the repair and regeneration of the CNS has received great research attention as such grafts can locally release therapeutics to the injury site, provide neuroprotection, and stimulate axonal regeneration [15,16]. These scaffolds have been precisely constructed to support the white matter tracts of the spinal cord, which has led to improvement in neuronal health and axonal regeneration directly as well as helped astrocytes to adopt a more conducive phenotype [17,18,19]. As reactive astrocytes can be favorably controlled, the formation of a glial scar can also be regulated.

To utilize a biomaterial-based strategy for SCI repair, it is essential to maintain the balance between the softness and mechanical strength of the biomaterial and to ensure its favorable environment. Several biomaterial characteristics, such as good biocompatibility and suitable porosity, permeability, and surface topography, which mimics the extracellular matrix (ECM) of the spinal cord, which is rich in glycosaminoglycan, are required [20,21,22]. Immediately after SCI, the ECM in the CNS begins losing its structural protein components, including collagen, which triggers glia cells to produce glycosaminoglycan, which is used as a gap-filling polysaccharide that maintains its own structure. However, an excessive glycosaminoglycan production as a result of lost structural protein components could lead to an enormous scar connective tissue formation that hinders neuronal regeneration. Different studies have used different biomaterials including natural or synthetic polymers and various methods to fabricate the scaffolds including electrospinning, freeze-drying, or molding to fill the damaged or gap area in SCI to maintain its structural integrity [23,24]. Based on this theory, we attempted to implant a collagen scaffold as a replacement structural protein to induce nerve growth and attenuate the excessive secretion of ECM factors during SCI. We hypothesized that this strategy could directly provide a new structural ECM into SCI to stabilize the structural integrity of the damaged area and be used as a repair method to reduce the formation of scar connective tissue by controlling astrocyte production for their normal functioning as a neural precursor cell type.

Collagen-based scaffolds are an attractive biomaterial used in tissue engineering and regenerative medicine owing to the inherent biological functionality, natural abundance, less antigenicity, and excellent biodegradability of collagen [25,26]. Several studies have demonstrated the great potential of collagen-based scaffolds as cell carriers and bridging material in numerous experimental animal models of CNS. The implantation has been demonstrated to be safe and has induced significant repair in SCI by increasing neurons and inhibiting glial scar formation [27,28,29]. Hence, collagen has become one of the most favored natural material sources for treating SCI. In this study, we developed a pure collagen scaffold and aimed to determine whether the neural plasticity can be manipulated by the implantation of the collagen scaffold in a rat SCI model.

## 2. Materials and Methods

### 2.1. Preparation of Collagen Scaffold

Collagen solution (0.25% *w*/*v*; C9791; Sigma-Aldrich, St. Louis, MO, USA) was prepared by dissolving type I collagen in 1% (*v*/*v*) acetic acid. Aliquots (0.275 mL) of the collagen solution were then loaded into glass vials. After being gradually cooled until reaching −20 °C overnight, the frozen aliquots were further transferred and processed in a freeze-drying chamber (FD12-2S; Kingming, Taipei, Taiwan) at −45 °C under a 300 mbar vacuum for 24 h, and then evenly ramped to 25 °C. Further, the collagen scaffold was treated with 0.5 mL of 2.5% (*w*/*v*) PCL/dichloromethane solution for 30 min in a closed vial before its lid was detached to allow solvent evaporation.

### 2.2. Physical Characteristics of the Collagen Scaffold

#### 2.2.1. Fourier Transform Infra-red (FTIR) Spectroscopy

The infrared spectra of the collagen scaffold were obtained using a FTIR spectrometer (Nicolet 8700, Thermo Scientific, Waltham, MA, USA) fitted with mercury-cadmium-telluride detector in the range of 400–4000 cm^−1^ at a resolution of 1 cm^−1^ and 32 scans/spectrum.

#### 2.2.2. Scanning Electron Microscopy (SEM)

The surface morphology of the collagen scaffold was examined with the help of a Hitachi S–3000N scanning electron microscope (Hitachi High Technologies, Krefeld, Germany). The samples were fastened to carbon stubs and mounted on aluminum stubs. SEM images were acquired under an accelerating voltage of 1.5 kV at a working distance of ~15.0 mm and at 500× to 1000× magnification.

### 2.3. In Vitro Biocompatibility

The collagen scaffold was sterilized using UV irradiation for 18 h before use. Human adipose stem cells (hASCs) provided by Dr. Cherng were briefly cultured in a medium consisted of keratinocyte serum-free medium (Life Technologies Ltd., Paisley, Scotland, UK) and 10% fetal bovine serum (Hyclone, Logan, UT, USA) under a humidified air containing 5% CO_2_ at 37 °C. hASCs at a density of 1 × 10^6^ cells cm^−2^ were seeded onto the collagen scaffold and incubated at 37 °C with 5% CO_2_ for 1 week, and the medium was changed every 2 days during incubation. The samples were further examined using immunocytochemical staining of beta-actin (β-actin) and octamer-binding protein 4 (OCT-4) markers counterstained with Hoechst 33342.

### 2.4. Rat Spinal Cord Injury Model

**Figure 1:**
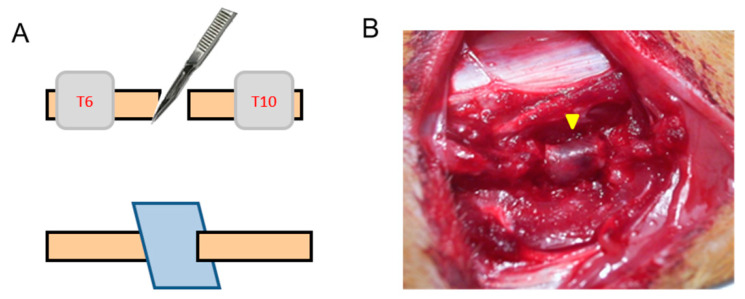
Rat spinal cord injury model. (**A**) An incision was made in the T8 of spinal cord, and the collagen scaffold (blue square) was inserted obliquely into the damaged area; (**B**) the position of implanted collagen scaffold in the T8 of spinal cord (yellow arrow).

Sprague–Dawley (SD) rats were obtained from BioLASCO Taiwan Co., Ltd., Taipei, Taiwan, R.O.C. A total of 18 adult SD rats with weights ranging from 240 to 280 g were used for the induction of SCI. Animal handling and experimental protocols were carefully reviewed and approved by the animal studies subcommittee of Animal Centre of the National Defense Medical Center, Taipei, Taiwan. (IACUC-17-066; IACUC-20-306). The rats were divided into four groups, as follows each: SCI-collagen scaffold (*n* = 3), SCI control (*n* = 3), normal spinal cord-collagen scaffold (*n* = 6), and normal control (*n* = 6). For developing the SCI model, the rats were anesthetized with intraperitoneal injection of xylazine (8 mg/kg) and ketamine (100 mg/kg), followed by a T8–10 laminectomy. The spinal cords of the rats were completely transected at T8 and a 5-mm-long piece of spinal cord tissue was removed. Next, the collagen scaffold was inserted obliquely into the damaged area (Figure 1). For developing the normal spinal cord–collagen scaffold model, the same laminectomy procedure was performed, and the spinal cord was explored without lesions, followed by covering its area with the collagen scaffold. Heating lamps were used near the cages during the first day post-surgery. The bladders were manually emptied twice daily. The rats were killed by intraperitoneally administering an overdose of sodium pentobarbital (≥100 mg/kg) at 1 and 4 weeks post-implantation. The spinal cord tissue was harvested and immediately stored in 10% neutral formalin.

### 2.5. Immunofluorescence Analysis

Both hASCs seeded in the collagen scaffold and the harvested spinal cord tissue were subjected to immunocytochemical/histochemical analyses. The samples were cryosectioned into 30 μm-thick slices and fixed with 4% paraformaldehyde, followed by the addition of 0.2% Triton X-100 solution. After 30 min, the sections were washed thrice with phosphate-buffered saline (PBS) for 5 min/wash and then blocked with 10% normal goat serum (Vector Laboratories Ltd., Burlingame, CA, USA). The primary antibodies including OCT-4 (polyclonal rabbit), anti-collagen (monoclonal mouse), anti-neurofilament (monoclonal rabbit), anti-glial fibrillary acidic protein (GFAP, monoclonal rabbit), anti-fibronectin (monoclonal rabbit), and anti-chondroitin sulfate (CS-56, monoclonal mouse) (all with 1:500 dilution, IgG type of antibody, Santa Cruz Laboratories, Dallas, TX, USA) were added to the samples and incubated for 2 h at 27 °C. The samples were then washed thrice with PBS for 5 min/wash. Furthermore, the secondary antibodies fluorescein isothiocyanate-conjugated anti-rabbit (Jackson ImmunoResearch, West Grove, PA, USA) and rhodamine-conjugated anti-mouse (AnaSpec, Fremont, CA, USA) with 1:1000 dilution were added to the samples and incubated for 1 h at 27 °C. In addition, the samples were stained with Hoechst 33342 (1:5000 dilution, AnaSpec, Fremont, CA, USA) for 15 min in order to visualize the nuclei. Fluorescent images were captured using a fluorescent microscope (Axio Lab.A1, Carl Zeiss AG, Oberkochen, Germany) embedded with a camera (Zeiss AxioCam ICm1, Carl Zeiss AG, Oberkochen, Germany). The sections were stained in triplicate, the staining being repeated three times.

## 3. Results and Discussion

### 3.1. Morphology and In Vitro Biocompatibility of the Collagen Scaffold

**Figure 2:**
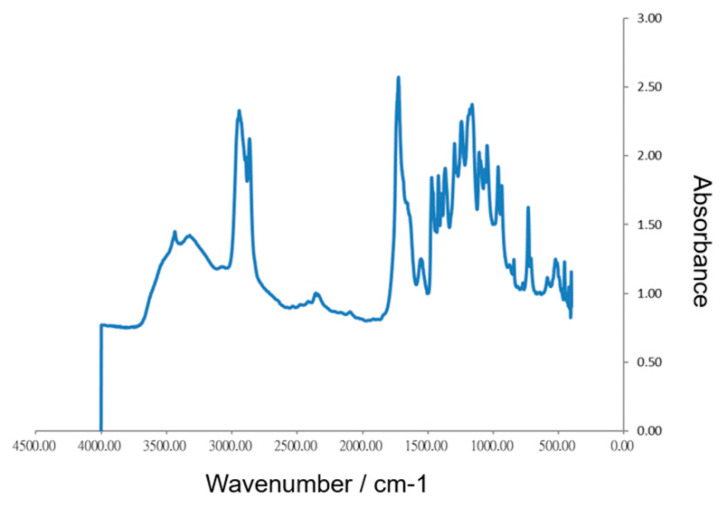
FTIR spectra of collagen scaffold.

The functional group characteristics of the collagen scaffold were identified using FTIR spectroscopy. As shown in Figure 2, the infrared spectrum of collagen scaffold displayed five major peaks, including peaks at 3500 and 3000 cm^−1^, corresponding to amide A and amide B, respectively, and three main bands for collagen at 1656–1644, 1560–1335, and 1240–670 cm^−1^ related to amide I, amide II, and amide III regions, respectively. Similar results have also been reported in previous studies [30,31].

**Figure 3:**
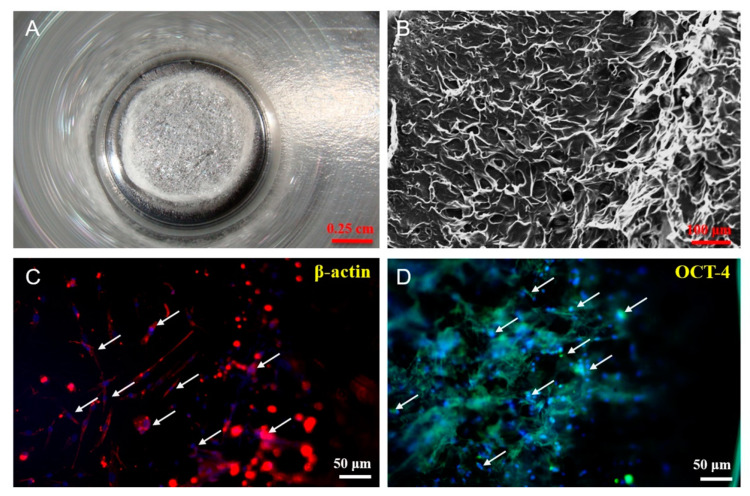
Characterization of the collagen scaffold. (**A**) Macroscopic appearance of collagen scaffold; (**B**) morphology of collagen scaffold characterized by SEM; (**C**,**D**) in vitro biocompatibility of collagen scaffold characterized by immunofluorescence staining of hASCs with beta-actin (β-actin) and octamer-binding protein 4 (OCT-4) counterstained with Hoechst 33342, respectively. (white arrow= cell nuclei stained by Hoechst 33342).

The functionality of regenerated tissue is considered highly dependent on the microstructure of the scaffold. As shown in Figure 3B, SEM revealed the morphology of the collagen membrane as a porous surface with pore sizes varying from approximately 50 to 100 μm, enabling cell adhesion and interchange of substances. Biocompatibility is an essential parameter for evaluating the capacity of a scaffold to generate successful cell-biomaterial interactions. Biological activities may be inadequate in regenerated tissue when the implanted scaffolds do not have suitable synergism with microenvironment [32]. Previous studies have demonstrated that cell proliferation and differentiation are promoted by collagen [33,34]. β-actin plays a critical role in most cellular processes such as cell cycle, cell migration, cell division, and immune responses, whereas OCT-4 is a substantial marker of self-renewal of cells [35,36]. In this study, hASCs were cultured within the collagen scaffold, and the immunofluorescent expression of β-actin and OCT-4 was evaluated. The scaffold was found to have excellent biocompatibility with hASCs, indicated by well-grown hASCs in the collagen scaffold (Hoechst 33342 expression) and positive expression of β-actin and OCT-4 staining (Figure 3C,D), thereby demonstrating its potential as a biomaterial for cell attachment and function.

### 3.2. In Vivo Biocompatibility and Immunofluorescence of the Implanted Collagen Scaffold in the Rat Spinal Cord Injury Model

To assess the biocompatibility and efficacy of the collagen scaffold for spinal cord repair in vivo, we developed a rat SCI model; the defects were either treated with implantation of the collagen scaffold or they remained untreated and served as a lesion control. Further, collagen scaffold-treated and untreated rats with normal spinal cord were also established as in vivo biocompatibility evaluation and control comparison.

**Figure 4:**
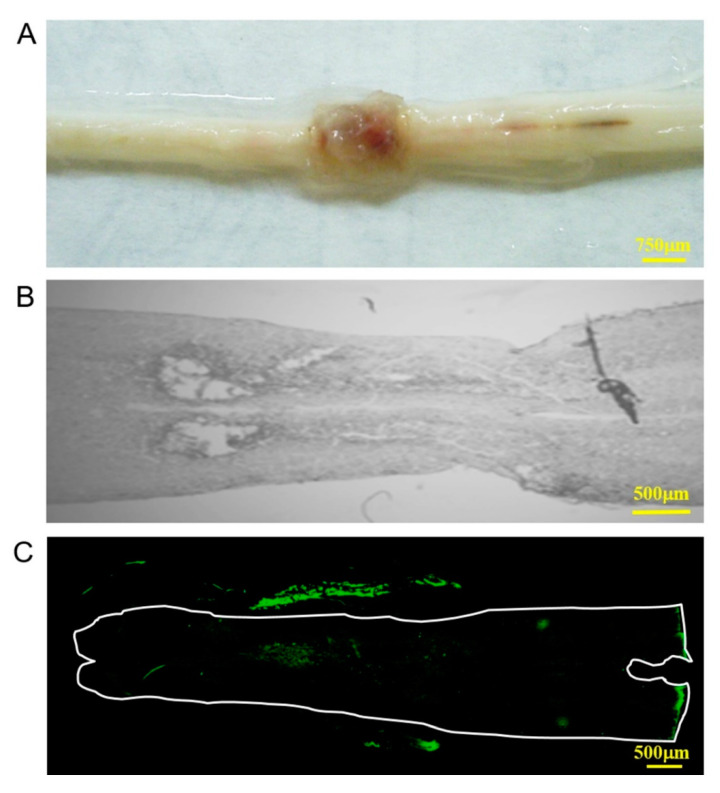
In vivo biocompatibility of the collagen scaffold implanted in the T8 of spinal cord for 4 weeks. (**A**,**B**) Macroscopic appearance and observation under a visible light microscope, respectively; (**C**) expression of anti-collagen antibody observed using immunofluorescence staining.

An important issue in the development of biocompatible scaffolds for tissue engineering is a negligible immune reaction and severe inflammatory response provoked by the scaffolds that might reduce healing or cause rejection by the body after implantation [37]. To evaluate the biocompatibility of the scaffold, it was obliquely inserted into the area of the T8 spinal cord and was examined 4 weeks after implantation. The scaffold was observed to adhere to the tissue membrane at the mimicking lesion site, and the area of the T8 spinal cord was observed without tissue damage and adverse foreign body reactions, including infection, bruising or edema, or inflammatory reactions (Figure 4A,B). This result demonstrates that the implantation was well accepted by the local spinal cord tissue, implying the outstanding biocompatibility of collagen scaffold. In addition, the positive expression of anti-collagen antibody in this implanted area (outside of the white line, Figure 4C) revealed that the collagen scaffold was biocompatible existed continuously for 4 weeks in the spinal cord tissue.

**Figure 5:**
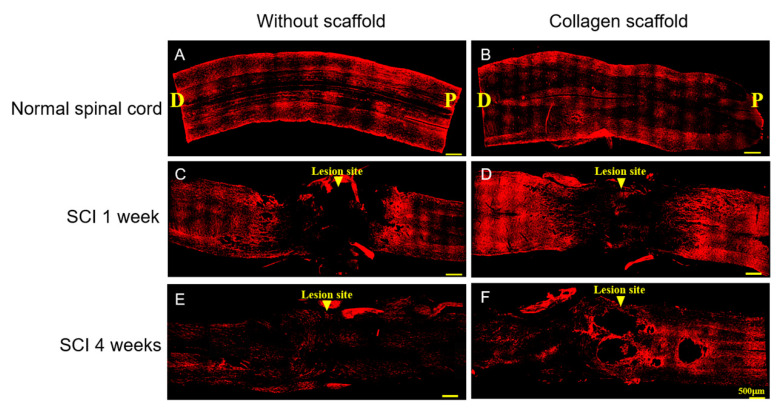
Immunofluorescence staining of neurofilament in the injured site of the spinal cord 1 week and 4 weeks post-implantation. (**A**,**B**) Rat normal spinal cord without and with collagen scaffold, respectively; (**C**,**D**) rat SCI model without and with collagen scaffold after 1 week post-implantation, respectively; (**E**,**F**) rat SCI model without and with collagen scaffold after 4 weeks post-implantation, respectively. (D = dorsal; P = posterior).

The effects of 1 and 4 weeks of collagen scaffold treatment in spinal cord injuries were further evaluated by immunofluorescence expression of neurofilament, GFAP, fibronectin, and CS-56. There was no different expression of neurofilament in the areas of injury in both treated and untreated groups at 1 week of implantation. At 4 weeks post-implantation, the injured spinal cord treated by implantation of the collagen scaffold showed notable neurofilament expression compared to the untreated group, and connection of tissues in the gap in the spinal cord was suspected to have occurred (Figure 5). Neurofilaments are major structural proteins that are expressed abundantly in the neurons of the CNS. They regulate axonal transport and signaling, and their leakage indicates the severity of neuronal loss in SCI [38,39]. Hence, it is suggested that the collagen scaffold could promote neuronal growth within the injured spinal cord.

**Figure 6:**
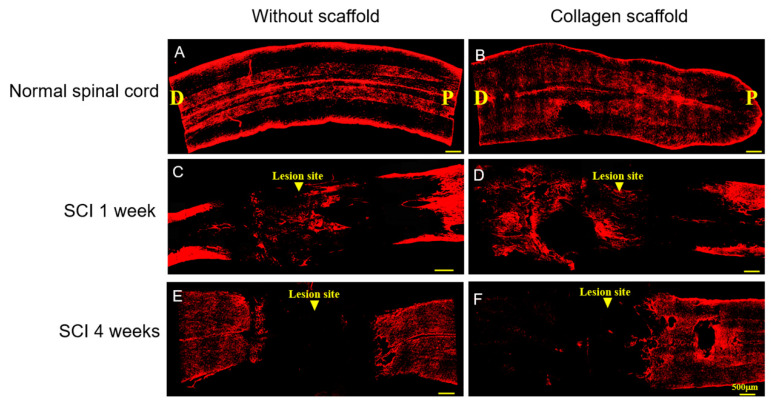
Immunofluorescence staining of glial fibrillary acidic protein (GFAP) in the injured site of the spinal cord 1 week and 4 weeks post-implantation. (**A**,**B**) Rat normal spinal cord without and with collagen scaffold, respectively; (**C**,**D**) rat SCI model without and with collagen scaffold after 1 week post-implantation, respectively; (**E**,**F**) rat SCI model without and with collagen scaffold after 4 weeks post-implantation, respectively. (D = dorsal; P = posterior)

Furthermore, we found that GFAP expression in both treated and untreated groups was notably high at 1 week post-implantation (Figure 6). At 4 weeks post-implantation, this expression was found to be decreased in the collagen scaffold treatment group, and nearly disappeared in the untreated group. GFAP is a responsible filament protein for the appropriate assembly and development of the cytoskeleton of astroglial cells, and its expression is remarkably upregulated by these cells during the acute phase of injury for activating astrocytes to produce various chemokines, cytokines, and growth factors [40,41]. After SCI, efficient regulation of astrocytes is important as these cells are essential for wound healing and supporting protective functions for neurons and oligodendrocytes [40]. However, under chronic SCI conditions, overexpression of GFAP is lethal because of the abundant deposition of GFAP-containing protein aggregates in astrocytes (glial scar), resulting in fatal neurodegeneration [42]. Our results thus indicate that the collagen scaffold could not only prevent chronically high GFAP levels that lead to glial scar formation but also maintain the production of astrocytes in a responsible manner for controlling their normal functions.

**Figure 7:**
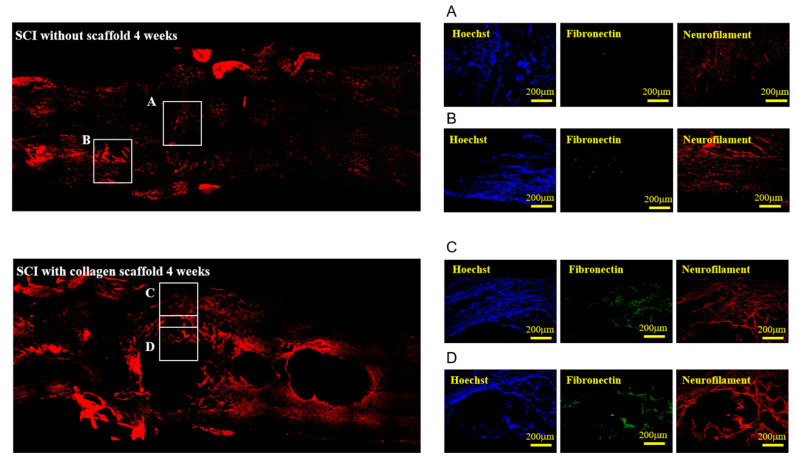
Immunofluorescence staining with Hoechst 33342, fibronectin, and neurofilament in the injured site of the spinal cord 1 and 4 weeks post-implantation. The enlarged images are provided as **A**, **B**, **C**, or **D** panel.

In particular, at 4 weeks post-implantation, we further analyzed the injured site of the spinal cord in both groups for fibronectin expression. Fibronectin expression was intensely high in the injured spinal cord treated by implantation of the collagen scaffold in comparison to the untreated group, while Hoechst 33342 expression, which represented the nuclei of the cells, in the treated group remained similar to that in the untreated group (Figure 7). Fibronectin is known to be an important component of the ECM that regulates neuronal activities, including neuroactive substance circulation and receptor activation, cell adhesion, and anti-neuroinflammation [43]. Studies demonstrated that fibronectin plays key roles in the extrasynaptic transmission and neuron–glia network and also exerts neuroprotective effects [43,44]. Therefore, our study demonstrated that collagen scaffold was likely to enhance the expression of fibronectin, into which axonal regeneration then occurred [45,46]. This evidence was further clarified with the expression of neurofilament, which corresponded to that of fibronectin (comparison between panel 7A and 7D that represented the same area in both groups). These results show that the abundance of neurons and axonal growth within the injured site is attributable to the implantation of collagen scaffold.

**Figure 8:**
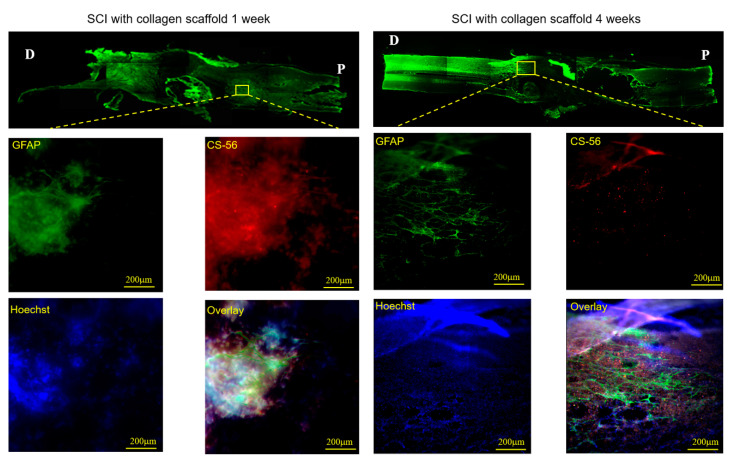
Immunofluorescence staining with Hoechst 33342, glial fibrillary acidic protein (GFAP), and anti-chondroitin sulfate antibody (CS-56) in the injured site of the spinal cord treated with collagen scaffold 1 week and 4 weeks post-implantation.

As the regenerative ability of the spinal cord is naturally poor after SCI, the occurrence of inhibitory factors such as cysts or glial scars that actively impede the restoration of function in the spinal cord should be prevented. Glial scars are comprised mainly of overactive astrocytes and the extracellular matrix molecule, chondroitin sulfate proteoglycan [47]. In this study, we investigated the expression of CS-56, a specific marker of chondroitin sulfate proteoglycan, in the collagen scaffold-treated SCI group (Figure 8). We found that the expression of SC-56 was remarkably decreased at 4 weeks post-implantation, suggesting that the collagen scaffold limited glial scar formation. This result corresponds with the reduced expression of GFAP, which represents controlled astrocyte production under collagen scaffold treatment.

Altogether, the results of this study confirm the ability of collagen scaffold in promoting spinal cord repair. The collagen scaffold used in the SCI rat model provided the desired structural proteins to the damaged tissue, limiting the unnecessary secretion of ECM molecules by astrocytes in the CNS environment for maintaining the structure of spinal cord tissue. Thus, this biological effect on astrocytes could prevent the occurrence of astrocyte hypertrophy and formation of nerve scars. Therefore, the structural proteins provided by the collagen scaffold applied in this study are beneficial for neuronal activities and manipulating synaptic plasticity, which in turn allows these neural precursor cells to differentiate into nerves and further achieve neural regeneration.

## 4. Conclusions

In summary, this study has demonstrated the feasibility of using collagen scaffold in SCI repair. The scaffold provided excellent structural proteins that manipulated the synaptic plasticity in the injured spinal cord, prevented the formation of glial scars, and promoted axonal regeneration, thereby indicating its excellent potential for clinical application in SCI. Thus, we suggest that if collagen scaffold generates a path or inhibits neuron growth in unexpected areas, it would improve the programming of nerve regeneration. According to this, we will supplement the collagen scaffold with the regulated stem cells or neural growth factors to optimize the function of nerve regeneration in future studies.
